# Analysis of factors influencing the dose of levothyroxine treatment in adequately controlled hypothyroid patients of different etiologies

**DOI:** 10.1016/j.heliyon.2024.e39639

**Published:** 2024-10-19

**Authors:** Márk Stempler, Bence Bakos, Tibor Solymosi, András Kiss, Richárd Levente Ármós, Balázs Szili, Szilvia Mészáros, Judit Tőke, Nikolette Szűcs, Péter Reismann, Pusztai Péter, Péter András Lakatos, István Takács

**Affiliations:** a Department of Internal Medicine and Oncology, Semmelweis University, 1083, Budapest, Korányi Sándor utca 2/a, Hungary; b Department of Internal Medicine and Haematology, Semmelweis University, Szentkirályi u. 46, Budapest, 1088, Hungary

**Keywords:** Hormone replacement, Levothyroxine, Autoimmune thyroiditis, Hashimoto's disease, Thyroidectomy

## Abstract

**Objective:**

The mainstay therapy of hypothyroidism is levothyroxine (LT4). In most cases lifelong treatment is warranted, therefore, choosing adequate doses are of paramount significance. The purpose of this study was to assess several factors that have been proposed to influence LT4 therapy including etiology of hypothyroidism, gender, age, bodyweight, BMI, concomitant drug use, disease severity and time since diagnosis in patients with stable, adequately controlled hypothyroidism.

**Methods:**

In this cross-sectional study we analysed past medical history, anthropometric data and biochemical parameters reflecting thyroid function of patients with chronic hypothyroidism who were adequately treated (TSH levels in normal range) with LT4 for at least 6 months. Potential predictors of LT4 requirement were evaluated using uni- and multivariate linear modelling.

**Results:**

191 individuals were enrolled in this study, who were divided into autoimmune (n = 147) and post-surgery (n = 44) groups. Mean age, time since diagnosis and LT4 dose (1.3 versus 1.1 mcg/kgBW) were significantly lower in the autoimmune group. In the post-surgery group age was the only significant (p = 0.016) predictor of LT4 dose. In the autoimmune group BMI (p = 0.001), time since diagnosis (p = 0.023), as well as their interaction (p = 0.012) turned out to be significant predictors of LT4 requirement.

**Conclusions:**

Our results implicate the necessity of differentiating between etiologies of hypothyroidism when starting or changing thyroxine replacement therapy. Patient in both groups required significantly lower doses of LT4 replacement, than previous reports suggest, to maintain stable euthyroidism. Distinctly different factors predicted hormone requirement in the two study groups.

## 1 Introduction

The estimated prevalence of overt hypothyroidism is approximately 0.1–2% in different populations [[1], [2], [3]], meanwhile the prevalence of subclinical disease can reach up to 7.5 % and be even more common with aging [4,5]. Not surprisingly levothyroxine (LT4), which is the mainstay of therapy, is among the most prescribed drugs in several developed countries [6,7]. Optimal hormone replacement is defined by long-term normalization of TSH and thyroid hormone levels. Since both under- and overtreatment carry potentially serious health risks [[8], [9], [10]], establishing optimal dosing regimens is of paramount importance. Appropriate LT4 replacement in overt hypothyroidism improves symptoms [11], quality of life [12], and life-expectancy [9], nevertheless, both under-, and overtreatment have been extensively reported [8,13]. It is disputed, whether patients with subclinical thyroid dysfunctions benefit from hormone therapy [14]. Various endogenous as well as exogenous factors are known to influence LT4 dosage including, but not limited to bodyweight, BMI, residual thyroid function, etiology of hypothyroidism and target TSH, age, gender, menopausal status [15], co-medications and co-existing diseases [16]. Recognizing these considerations in the treatment of primary hypothyroidism is helpful in individualizing LT4 dose at the time of starting the patients off on medication. Given that the majority of hypothyroid patients are in need of lifelong hormone substitution, optimizing follow-up frequency could lessen the burden of the disease for the patient, the physician, as well as for the healthcare system. The use of these predictors, however, is somewhat arbitrary in everyday clinical practice due to the scarcity and often contradictory nature of the available data.

In clinical practice, bodyweight is the most well established and easiest to use factor. Current guidelines recommend an LT4 dose of 1.6–1.8 mcg/kgBW in overt hypothyroidism and 0.8–1 mcg/KgBW for subclinical hypothyroidism [11]. This dosing was shown to be adequate only in approximately one third of patients [2,17], which points towards the significance of other factors at play. It is important to note, however, that these doses are based on a limited number of trials with a relatively small total number of participants [[18], [19], [20]]. Almost equally as important is the fact that patients in these studies were evaluated shortly after total or near-total thyroidectomy. Data regarding optimal LT4 dosing in other contexts, such as Hashimoto's thyroiditis is scarce [20].

The effect of BMI is the second most frequently cited determinant of LT4 requirement [19,21,22]**.** Increased BMI leads to a decrease in the per kilogram dosage requirement. The available data once again comes mostly from patients who underwent near-total thyroidectomy. Guidelines concerning patients with chronic autoimmune thyroiditis are mostly based on expert opinion as even observational data is surprisingly scarce [20,23]. For instance, papers discussing the treatment of Hashimoto's acknowledge the fact that due to the progressive destruction of thyreocytes, patients' LT4 demand could increase over time, however, no data is available on how the hormone substitution requirement changes throughout the years.

There is also an established decrease in LT4 requirement among elderly patients with hypothyroidism. Reduced muscle-mass, impaired degradation- and excretion of thyroid hormones have all been suggested as a potential cause [[24], [25], [26]]. Some other studies, however, have proposed, that the effect of age is primarily explained by age-related weight change.

Papers focusing on the effect of certain co-medications, from which gastric acid reducers are emphasised due to their widespread use, are mostly small case series [[27], [28], [29]] and retrospective studies [30].

The role of gender in thyroid hormone metabolism is also debated. Estrogen therapy and testosterone replacement have both been shown to impact levothyroxine requirements due to their opposing effects on TBG levels [31,32]. The effect of endogenous sexual steroids, however, is much less clear. The role of anti-thyroid peroxidase antibodies (aTPO) is disputed as well. It has been previously reported that the seronegative form of the disease is generally associated with milder presentation and lower LT4 requirements [33]. Starting LT4 therapy could potentially lower antibody levels by itself [34,35]. As the autoimmune destruction of thyroid tissue progresses, aTPO levels might decrease or even normalise, thus theoretically seropositive cases could become seronegative. In seropositive cases, there are also some reports suggesting an association between autoantibody titers, LT4 doses and symptomatology [33,36].

In this present study, we aimed to assess and quantify determinants of LT4 dose in patients with primary hypothyroidism of various etiologies, in whom stable euthyroid state was clinically as well as biochemically confirmed for at least 6 months. Our main focus was Hashimoto's thyroiditis with special attention to time since diagnosis and to BMI.

## 2 Methods

### 2.1 Patients and study design

In this cross-sectional study we gathered data from all hypothyroid patients presenting at our institution's endocrinology outpatient clinics in a one-year period between August 2021 and August 2022.

### 2.2 Inclusion criteria

- documented primary hypothyroidism treated with LT4 monotherapy for at least 2 years
- TSH within normal range (0.4–4.5 mU/L) for at least 6 months, both at the time of screening and at the time of the previous visit
- age over 18 years
- capacity to give written informed consent

### 2.3 Exclusion criteria

Individuals with comorbidities that could significantly alter thyroxine metabolism.

- gastrointestinal conditions associated with malabsorption (eg. celiac disease)
- chronic kidney failure (eGFR<50 mL/min/1.73 m^2^)
- chronic liver disease (GOT, GPT, GGT, ALP, Bi levels three times greater than normal upper range)
- congestive heart failure
- active malignancies
- co-occurring endocrinopathies (eg. adrenal insufficiency)
- age under 18 years
- calcium-carbonate antacid use
- incapability or unwillingness to give written informed consent

Case definition of autoimmune hypothyroidism was based on a history of either overt hypothyroidism or repeated findings of subclinical hypothyroidism, along with one or more of the following markers of autoimmune inflammation: elevated aTPO levels, elevated aTG levels, ultrasound changes indicative of inflammation or cytological results showing inflammation. Case definition for post-surgical hypothyroidism was based on a history of total or near-total thyroidectomy. Patients in this group were generally started on hormone substitution right after surgery, thus documented TSH elevation was not part of our criteria for establishing the diagnosis. Individuals on thyroxine substitution who underwent only lobectomy were not included.

We reviewed disease history including etiology of hypothyroidism, initial TSH at the time of diagnosis, concomitant medications and comorbidities, gathered anthropometric data, and measured biomarkers of thyroid function including TSH, FT4, and aTPO. All enrolled patients underwent thorough physical examination by one of the physicians in our research team. The enrolled patient population presented no specific signs or symptoms of hypothyroidism.

Patients gave written informed consent before entering the study and prior to any study related procedures. The consent extended to the research team accessing their relevant medical history, anthropometric-, and laboratory data. This work was approved by the Hungarian National Scientific and Ethics Committee of the Hungarian Medical Research Council (approval number: 38233-1/2019/EKU), and was carried out in accordance with the World Medical Association's Declaration of Helsinki. Patients laboratory samples were measured on the same day of the visit and were processed within 2 h.

### 2.4 Laboratory testing

We performed third generation TSH testing, and free thyroid hormone (FT4) level measurements using chemiluminescence immunometric assays on the Atellica IM analyzer (Siemens Healthcare Diagnostics Inc., Erlagen, Germany). Measuring range for these methods were 0.008–150.0 mIU/L and 1.3–154.8 pmol/L for TSH and FT4 respectively. Calibration of the measurement of FT4 and TSH was made by internal standard manufactured using USP material, Calibrator A (CAL A) and WHO 3rd International Standard for human TSH (IRP 81/565) respectively. We measured anti-TPO levels using an electrochemiluminescence immunometric assay on the Roche Cobas e, platform (Roche Diagnostics, Mannheim, Germany) with a measuring range of 9–600 IU/mL. For control Liquicheck Immunoassay Plus Control (BIO-RAD, reference number:360) was used. All parameters were measured in the central laboratory of Semmelweis University. Patient were asked to withhold LT4 supplementation in the morning of blood sampling.

### 2.5 Statistical analysis

Means (M) ± standard deviations (SDs), medians (Mdn), and interquartile ranges (IQR) are reported for continuous variables. Categorical variables are presented as counts and percentages. Normality and homogeneity of variances (assumption that the variance within each sample is equal for all samples) were tested for using the Shapiro–Wilk test and Levene's test respectively. In cases where assumptions were possibly violated we used robust corrections such as bootstrapped (bias corrected and accelerated - BCa) confidence intervals and heteroscedasticity-consistent standard errors. Between-group differences in continuous and categorical outcomes were assessed using *t*-test or ANOVA and Chi-square or Fisher's exact test respectively. Factors with a potential influence on LT4 requirement were assessed separately by etiology of hypothyroidism (autoimmune vs. post-surgery) in three consecutive steps. Firstly, all independent variables were tested separately in a univariate linear regression model with LT4 dose (mcg/bodyweight kg) as the dependent variable. Predictors showing significant effect were entered in tandem in a multivariate model. Variables retaining a significant effect after this correction were tested for potential higher-level interactions. Multiple testing was corrected for using the Bonferroni method. Statistical significance was considered with two-sided p values < 0.05. All statistical calculations were performed with IBM SPSS Statistics for Windows version 28.0 (Released 2021, IBM Corp, Armonk, NY).

## 3 Results

One hundred and ninety-one participants were eligible to this study. Patient characteristics by etiology of hypothyroidism are summarized in Table 1. Every participant's TSH value were in the normal range (mean value in the autoimmune group: 2.0 ± 1.00 mIU/L and in the post-surgery group: 1.47 ± 0.67 mIU/L). Seventy-seven percent of the patients had autoimmune thyroiditis, meanwhile 23 % underwent thyroidectomy earlier. As expected, both groups consisted of dominantly females (92 % and 86 % respectively). Mean age was significantly higher is the post-surgery group (47.7 ± 15.0 years versus 53.6 ± 12.5 years). BMI in both groups were comparable (26.6 ± 5.9 kg/m^2^ versus 27.1 ± 5.5 kg/m^2^). Thyroidectomised patients had significantly longer time since diagnosis (14.1 ± 11.1 years versus 10.2 ± 6.4 years). Only 17 patients (9 %) were taking gastric acid reducing therapy. Bodyweight adjusted LT4 doses were significantly higher in the post-surgery group (**1.27 ± 0.38** mcg/kgBW versus **1.1 ± 0.4** mcg/kgBW).

**Table 1:** Patient characteristics by etiology of hypothyroidism.

Number of patients	Autoimmune	Post-surgery	p
	147 (77 %)	44 (23 %)	
Gender of patients			0.375
female	135 (92 %)	38 (86 %)	
male	12 (8 %)	6 (14 %)	
Age (years)			**0.011**
M±SD	47.7 ± 15.0	53.6 ± 12.5	
Mdn	45	53	
IQR	24	16	
Bodyweight (kg)		74.6 ± 16.1	0.907
M±SD	74.7 ± 17.6		
Mdn	70		
IQR	22.5		
BMI (kg/m^2^)			0.590
M±SD	26.6 ± 5.9	27.1 ± 5.5	
Mdn	25	26,4	
IQR	6.7	7.8	
time since diagnosis (years)			**0.018**
M±SD	10.2 ± 6.4	14.1 ± 11.1	
Mdn	8	11	
IQR	9	16	
PPI co-medication	12 (8 %)	5 (11 %)	0.548
aTPO (IU/mL)			
M±SD	326.9 ± 844.2		
Mdn	97		
IQR	318		
aTPO seronegative patients	52 (35 %)		
LT4 dose (mcg)			**0.019**
M±SD	80.2 ± 29.4	94.7 ± 33.9	
Mdn	75	100	
IQR	50	50	
LT4 dose/KgBW (mcg/kg)			**0.013**
M±SD	1.1 ± 0.4	1.27 ± 0.38	
Mdn	1.1	1.3	
IQR	0.6	0.4	

In the post-surgery group, the univariate analysis showed that only age (F = 6.61, β = −0.011, **p = 0.011**) affected LT4 dose (mcg/kgBW) significantly. A negative correlation was observed, meaning that LT4 requirement decreases with increasing age. BMI (F = 1.53, β = −0.013, p = 0.101), gender (t(42) = -1.415, B = −0.23, p = 0.164), time since diagnosis (F = 1.41, β = 0.006 p = 0.101) and concomitant PPI/H2-antagonist therapy (t(42) = -0.262, B = −0.048, p = 0.795) had no effect, however, the number of patients receiving these medications were rather small. These results are summarized in Table 2.

**Table 2:** Univariate linear models with predictors of LT4 dose (mcg/kgBW) in post-surgery patients. In this patient group only age has proven to be a significant determinant of thyroxine dose, with mean thyroxine requirement decreasing by 0.11 mcg/kgBW for every year.

Predictor	F	r^2^	B ± SE	p
**Age (years)**	**6.613**	**0.136**	−0.11 ± 0.004	**0.011**
Time since diagnosis (years)	1.415	0.33	0.006 ± 0.005	0.101
Bodyweight (kg)	0.086	0.002	−0.001 ± 0.004	0.771
BMI (kg/m^2^)	1.531	0.035	−0.013 ± 0.011	0.101

Among participants with Hashimoto's thyroiditis BMI (F(1.145) = 11.27, β = −0.27, **p = 0.001**) showed a negative-, and time since diagnosis (F(1.145) = 8.05, β = 0.014, p = **0.015**) a positive correlation with LT4 requirement (mcg/kgBW). Specifically, higher BMI values were associated with lower LT4 doses per kgBW, while a longer disease course was associated with increased mean thyroxine requirement. Age (F(1.145) = 0.03, β = −0.01, p = 0.82), gender (t(145) = 0.89, B = 0.10, p = 0.317), and aTPO levels (F(1.143) = 0.08, β = 0.002, p = 0.66) had no effect in the univariate analysis. Thirty-five percent of the autoimmune group were aTPO seronegative. The concomitant use of acid-reducers was not associated with a significant difference in LT4 doses (t(145) = -1.76, B = −0.21, p = 0.057), however, this could be due to the relatively low number of participants receiving PPI or H2-antagonist treatment.

**Fig. 1:**
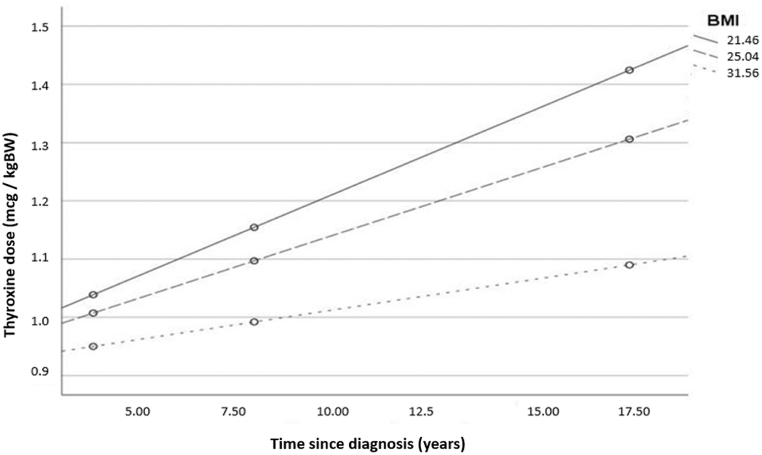
The effect of time since diagnosis on LT4 requirement as a function of BMI in patients with Hashimoto's thyroiditis. (Regression lines are shown for the 16th, 50th, and 84th BMI percentiles of our sample). The interaction between the two predictors was shown to be significant in the moderation analysis.

Both BMI (β = −0.33, **p = 0.001**) and time since diagnosis (β = −0.30, **p = 0.002**) retained its significance when entered simultaneously in a multivariate model (F(2.144) = 13.39, r^2^ = 0.16, **p < 0.01**). Moderation analysis revealed a significant BMI x time since diagnosis interaction (**p = 0.012**) that is shown in Fig. 1.

Data on the severity of hypothyroidism at the time of diagnosis was available for only 53 % of the Hashimoto's cases. In this subgroup we construed a model with the use of initial TSH (TSH>10mIU/L vs TSH<10mIU/L) as an additional predictor. Patients with an initial TSH value < 10mIU/L required significantly lower doses of LT4. This model is shown in Table 3.

**Table 3:** Multivariate linear model with predictors of LT4 dose (mcg/kgBW) in patients with Hashimoto's thyroiditis. F(4.72) = 7.57, p < 0.001, r^2^ = 0.3 for the whole model. Time since diagnosis and initial TSH have both shown a significant positive correlation with thyroxine dose. BMI also had a significant positive effect that was moderated by time since diagnosis (see also Fig. 1.).

Predictor	B ± SE	p	η^2^
Intercept	0.53 ± 0.32	0.33	0.014
Initial TSH (TSH<10 vs TSH>10)	0.23 ± 0.08	**0.009**	0.1
BMI (kg/m^2^)	0.01 ± 0.02	0.623	0.004
Time since diagnosis (years)	0.15 ± 0.05	**0.006**	0.094
BMI x time since diagnosis interaction	−0.005 ± 0.002	**0.015**	0.068

## 4 Discussion

We analysed the effect of several factors on LT4 therapy, which have been proposed to influence LT4 doses in earlier studies. In addition, our selection criteria ensured the enrolment of only stably controlled hypothyroid patients (diagnosis at least over 2 years, euthyroidism with no LT4 dose modification for at least half a year).

We found that mean LT4 requirement among patients who underwent total (or near-total) thyroidectomy was notably lower than the aforementioned 1.6–1.8 mcg/kgBW. Only 5 (11 %) patients received doses at or above 1.6 mcg/kgBW. This could be a result of significant differences in the patients’ characteristics compared to earlier studies. Longer time since diagnosis, older age and differences in the extent of thyroidectomy stand out as probable causal factors. Another explanation might be that our enrolled patients were in relatively good health, individuals with comorbidities, which could modify (in this case increase) LT4 doses were excluded from the study. Our data shows a mean of 1.3 mcg/kgBW LT4 dose as an optimal estimation of hormone replacement requirement for patients who underwent total thyroidectomy, which is markedly lower than the recommendation of current guidelines [11].

Among our patients with Hashimoto's thyroiditis, the mean LT4 dose was 1.1 mcg/kgBW, consistent with findings by Croce et al. [37]. The generally accepted rationale for lower LT4 requirements in this group is the presence of residual endogenous hormone synthesis.

While it is widely acknowledged that LT4 dose requirements tend to increase as Hashimoto's disease progresses, the underlying reasons remain debated. The most widely supported theory is that organ-specific autoimmune inflammation gradually destroys healthy thyroid tissue, ultimately halting endogenous hormone production [38]. Some authors also suggest that progressive alterations in peripheral thyroid hormone metabolism might play a role [39], though this explanation is more controversial [11,40].

The exact relationship between time since diagnosis and LT4 requirements is not well defined and has been infrequently studied. In our study time since diagnosis was associated with a 1.3 mcg/year or a 0.14 mcg/kgBW/year mean increase in thyroxine requirements. This effect, however, was significantly moderated by BMI as discussed below.

Patients with a TSH below 10 mIU/L at the time of diagnosis are considered to be in an earlier stage of the disease [41,42]. Comparing these individuals to those with more advanced hypothyroidism at diagnosis allows for the consideration of the effect of pre-diagnosis disease duration. In our study, initial TSH showed a significant association with current LT4 doses. Individuals with TSH<10mIU/L at diagnosis required, on average, 0.23 mcg/kgBW less LT4, independent of known time since diagnosis. In our final model, initial TSH had the largest effect size, significantly improving model accuracy. These results further emphasize the role of disease duration - both known and unknown - in determining the LT4 requirement of Hashimoto's patients.

BMI has been shown to affect levothyroxine requirements independently of bodyweight in individuals after thyroidectomy [21,43], however, this effect was not statistically significant in our study population.

Compared to post-surgery patients, the effect of BMI (the higher it is the less per kgBW is required) was more pronounced in our sample of Hashimoto's patients, and remained significant even in the multivariate analysis. There was also a significant interaction between BMI and time since diagnosis showing that the effect of time since diagnosis was meaningful primarily in individuals with lower BMI, and decreased significantly in obese patients. This is probably the result of LT4 requirements increasing much earlier, thus plateauing also earlier in the course of the disease in overweight patients.

Bodyweight-adjusted LT4 doses were significantly inversely correlated with age among our patients in the post-surgery group. This suggests that the age-related decrease in LT4 requirement is not solely a consequence of changes in bodyweight or body-composition. Our finding on age-related LT4 dose decrease is supported by a recently published longitudinal study from Gavigan et al. [44], which revealed that 84 % of individuals over the age of 65 years who were on LT4 substitution, were euthyroid on a dose of less than 1.6 mcg/kgBW. Age did not turn out to be a significant predictor of LT4 dose in the autoimmune group. A possible explanation could lay in the individual variety of the initiation and of the progressivity of the disease. Even though Hashimoto's disease typically manifests at younger ages, it can develop later in life as well.

Several potential predictors showed no significant association with LT4 dosage, including the use of gastric acid reducers, gender, and aTPO levels.

Existing data on the effects of PPIs and H2-antagonists on thyroxine absorption and LT4 requirements are derived from a series of small-scale studies, which may explain the contradictory findings and lack of consensus in this area. Our findings align with prior research showing no correlation between the use of these drugs and thyroxine dosage. No patient was taking calcium carbonate at the time of screening. However, the relatively small number of participants on acid reducers limits the generalizability of our results. Additionally, our study did not compare different LT4 formulations, which may vary in bioavailability at different gastric pH levels [29].

The prevailing pathogenic model of Hashimoto's thyroiditis focuses on T-cell-mediated destruction of thyrocytes, with humoral mechanisms considered secondary. Nevertheless, some earlier studies have indicated a potential association between elevated aTPO levels and greater LT4 requirements [33]. However, our data showed no correlation between aTPO levels and LT4 dosage. The prevalence of aTPO seronegativity among patients with chronic autoimmune thyroiditis remains unclear, though some estimates are available in the literature [37,45]. In our autoimmune group, 35 % of patients had aTPO levels below 30 U/mL.

The primary strengths of this study are its relatively large sample size and the exclusion of patients with potential confounding comorbidities. Additionally, the long average time since diagnosis, verifiably stable and biochemically sufficient LT4 replacement, and the separate analysis of predictors based on etiology enhance the generalizability of our findings for everyday clinical practice.

Our study has several limitations, chief among them being its cross-sectional design, which necessitates further validation of our results through longitudinal studies. Although all patients in the autoimmune group verifiably had either subclinical or overt hypothyroidism throughout the course of their disease, data on the initial elevated TSH level were available for only 53 % of participants. Additionally, the exclusion of patients with recent changes in levothyroxine dose may introduce selection bias, potentially overrepresenting individuals with higher LT4 requirements among those excluded. We also observed a significant gender imbalance in our sample. This is unsurprising given the higher prevalence of thyroid disease in females, yet may represent another source of bias.

## 5 Conclusion

In this study, we reported predictors of levothyroxine replacement in a cohort of 191 individuals with chronic hypothyroidism. Among patients who had undergone total or near-total thyroidectomy, mean bodyweight-adjusted LT4 doses were 1.3 mcg/kgBW - surprisingly lower than anticipated based on current guidelines. In this group, patient age emerged as the primary predictor of thyroxine dose.

For those with autoimmune thyroiditis, the mean LT4 replacement dose was 1.1 mcg/kgBW, with both BMI and time since diagnosis significantly influencing bodyweight-adjusted LT4 requirements. The interaction between these two factors suggests that individuals with normal or low BMI may require more frequent dose adjustments over the course of the disease.

## CRediT authorship contribution statement

**Márk Stempler:** Writing – review & editing, Writing – original draft, Visualization, Validation, Project administration, Methodology, Investigation, Formal analysis, Data curation, Conceptualization. **Bence Bakos:** Writing – review & editing, Writing – original draft, Project administration, Methodology, Formal analysis, Conceptualization. **Tibor Solymosi:** Investigation, Data curation. **András Kiss:** Investigation, Data curation. **Richárd Levente Ármós:** Investigation, Conceptualization. **Balázs Szili:** Data curation. **Szilvia Mészáros:** Data curation. **Judit Tőke:** Data curation. **Nikolette Szűcs:** Data curation. **Péter Reismann:** Data curation. **Pusztai Péter:** Data curation. **Péter András Lakatos:** Writing – review & editing, Visualization, Conceptualization. **István Takács:** Writing – review & editing, Visualization, Supervision, Project administration, Methodology, Conceptualization.

## Ethics approval

This work was approved by the Hungarian National Scientific and Ethics Committee of the Hungarian Medical Research Council (approval number: 38233-1/2019/EKU), and was carried out in accordance with the World Medical Association's Declaration of Helsinki.

## Consent for publication

Written informed consent was obtained from all patients enrolled in this study. A copy of the written consent is available for review by the Editor-in-Chief of this journal upon request.

## Data availability

This study's data has not been deposited into a publicly available repository, however, it will be made available on request.

## Sources of funding

This research did not receive any specific grant from funding agencies in the public, commercial, or not-for-profit sectors.

## Declaration of competing interest

The authors declare that they have no known competing financial interests or personal relationships that could have appeared to influence the work reported in this paper.
